# A study of the relationship between social anxiety and mask-wearing intention among college students in the post-COVID-19 era: mediating effects of self-identity, impression management, and avoidance

**DOI:** 10.3389/fpsyg.2023.1287115

**Published:** 2023-11-21

**Authors:** Tiansheng Xia, Xuan Xu, Shiyao Ding

**Affiliations:** ^1^School of Art and Design, Guangdong University of Technology, Guangzhou, China; ^2^Guangdong International Center of Advanced Design, Guangdong University of Technology, Guangzhou, China

**Keywords:** social anxiety, mask-wearing intention, college students, impression management, avoidance, self-identity

## Abstract

**Introduction:**

During the 2019 coronavirus (COVID-19) pandemic, wearing masks not only prevented transmission of the virus but also reduced social anxiety to some extent. With the end of the epidemic, the intention to wear masks to prevent transmission declined, but the effect of social anxiety on the intention to wear masks is unclear. The current study investigated the effects of social anxiety and fear of COVID-19 on mask-wearing intentions in the post-epidemic era, using self-identity, impression management and avoidance as mediating variables.

**Methods:**

In total, 223 college students participated in the current study, and the related variables were measured using the social anxiety scale, the social behavior questionnaire, the self-identity questionnaire, and the mask-wearing intention questionnaire.

**Results:**

The results showed that social anxiety was significantly positively correlated with avoidance, impression management, and intention to wear masks, and significantly negatively correlated with self-identity. The fear of COVID-19, avoidance, and impression management were significantly positively correlated with mask-wearing intentions, while self-identity was significantly negatively correlated with mask-wearing intentions. Social anxiety affected college students’ intention to wear masks through three main pathways: the mediating role of avoidance, impression management, and the chain mediating role of self-identity and avoidance. The fear of COVID-19 directly and positively affected mask-wearing intentions.

**Discussion:**

The current study reveals the differential pathways of the effects of COVID-19 fear and social anxiety on mask-wearing intentions in the post-COVID-19 era, and the findings have some practical implications for social anxiety interventions.

## 1 Introduction

During the 2019 coronavirus (COVID-19) epidemic, numerous precautionary measures were recommended by the health authorities to avoid the spread of the virus, such as keeping a social distance and wearing masks, which had a significant impact on people’s lives and social interactions (Saladino et al., 2020; Litwinowicz et al., 2022; Sukul et al., 2022). Studies have shown that COVID-19 led to profound psychological and behavioral changes in college students (Alemany-Arrebola et al., 2020; Ma et al., 2020). The epidemic limited social interactions, and, for a large portion of the time, most college students were unable to access places where they interacted face-to-face with peers, faculty members, and others, especially on campus and in indoor public spaces (Barankevich and Loebach, 2022). Previous research has found that although the direct impact of wearing masks during the first social interaction may be quite small, it is still disruptive (Crandall et al., 2022). With the advent of the post-COVID-19 era, countries are gradually removing regulations related to mandatory mask-wearing and no longer require masks to be worn in public. The anti-epidemic situation has entered a phase of normalization, but there is always the possibility of small-scale outbreaks of localized epidemics, and vigilance against epidemics still cannot be relaxed. Research on whether people will reduce mask-wearing in response to changes in policy toward openness or continue to wear masks for hygienic reasons or social psychology could help to explain individual behavioral and psychological changes in the post-COVID-19 era.

Social anxiety (SA) has been identified as a risk factor for future health problems among college students. The global prevalence of social anxiety among college students is about 7–33% (Honnekeri et al., 2017; Jaiswal et al., 2020), and social anxiety is as prevalent among college students in the United States as it is in the general population (Schry et al., 2012). Likewise, in China, social anxiety is one of the serious public health problems in the college student population (Sun et al., 2019; Xia et al., 2023). If social anxiety is not ameliorated or corrected, it may develop into severe social anxiety disorder (SAD) and negatively affect college students’ academic performance, academic achievement and social relationships (Brook and Willoughby, 2015). According to an exploratory review of the effects of mask-wearing on social anxiety (Saint and Moscovitch, 2021), in the context of an epidemic, mask-wearing may be perceived as a social norm, with behaviors being influenced more by perceived norms than by the perceived protective value of the mask (Nakayachi et al., 2020); while mask-wearing can also be perceived as a self-concealing behavior. Thus researchers believe that wearing masks relieves anxiety and promotes mask use, a finding that suggests that people think more about subjective feelings than objective risks (Nakayachi et al., 2020). Previous studies have scrutinized the role of mask-wearing in clinical applications and explored the perceptions of mask-wearing (Howard, 2021; Luo et al., 2021; Schneider and Leonard, 2022; Vann et al., 2022). However, the other psychological and sociological mechanisms behind mask-wearing remain largely unexamined. Therefore, in the post-COVID-19 context, the present study aimed to explore the differences in the mechanisms between the two, and how the fear of COVID-19 or social anxiety has an impact on mask-wearing intentions when wearing masks is no longer defined as a social norm, and wearing or not wearing masks has become a matter of personal choice.

### 1.1 Fear of COVID-19 and mask-wearing intentions

Fear was prevalent among patients, healthcare workers and the public during the COVID-19 epidemic (Liu et al., 2020). Previous research has shown that fear of an epidemic affects psychological responses and behavior (Di Crosta et al., 2020). The World Health Organization recommends wearing face masks in public places because there is evidence that wearing masks is effective in controlling the spread of COVID-19 (Howard et al., 2021; Rader et al., 2021). In addition, researchers in Taiwan revealed the importance of fear as a driver of public adherence to protective measures during the COVID-19 pandemic (Chin et al., 2021), and the mechanism may be that fear induces vigilance against personal threats, which leads individuals to take measures to protect themselves from harm (Witte and Allen, 2000). Wearing masks can make people to feel safer (Cartaud et al., 2020). Regardless of the actual ability of masks to prevent infections, wearing masks can alleviate people’s anxiety (Nakayachi et al., 2020). Currently, although COVID-19 has greatly abated, the behavioral and psychological effects of the outbreak on people still exist. Fear of COVID-19 significantly predicts people’s preventive behaviors (Frounfelker et al., 2021; Roberto et al., 2021).

People may be inclined to continue wearing masks to ward off the disease if they perceive that COVID-19 still poses a possible threat to their personal health. Therefore, this study proposed Hypothesis:

*H1*: Fear of COVID-19 positively influences college students’ mask-wearing intentions.

### 1.2 Social anxiety and mask-wearing intentions

Social anxiety is fundamentally driven by the fear that one’s appearance or behavior does not conform to societal expectations and norms (Clark and Wells, 1995; Rapee and Heimberg, 1997). According to Baldwin and Main (2001), underlying social anxiety is the “fear of judgment,” where people worry about what others think of them, especially negatively. The YouGov UK Mask Wearership Survey, conducted in May 2020, found that non-mask-wearers were more likely to feel a lack of self-consciousness and to worry about being judged negatively compared with mask-wearers (Smith, 2020). Sim et al. (2014) highlighted the benefits and effects of wearing masks in their study, including discomfort and embarrassment for the individual. The findings of Warnock-Parkes et al. (2021) suggested that it is not only health-related beliefs that lead to mask-wearing, but also the thoughts that people have when wearing masks (e.g., looking confident, looking anxious, etc.) also predicted mask use. Additionally, a report during the COVID-19 epidemic mentioned that wearing a mask was seen as a “security blanket” (Ray, 2020), which, in part, alleviated social pressure due to a fear of exposing cosmetic flaws or anxious behavior (Ray, 2020; Sloat, 2020). Thus, the fear associated with negative judgments in social interactions in socially anxious people may be closely related to their intentions and behavior regarding mask-wearing. Therefore, the present study aimed to investigate the factors that influence the relationship between social anxiety and the intention to wear masks in order to elucidate the mechanisms of their influence.

#### 1.2.1 Avoidance

According to the cognitive model of SA, safety behavior is a self-protective strategy that people use to prevent exposure to a feared outcome (Goetz et al., 2016), and it is one of the most important ways that socially anxious people reduce their anxiety. Research usually classifies safety behaviors into two types (Plasencia et al., 2011; Gray et al., 2019), namely avoidance and impression management. Avoidance refers to behaviors that attempt to hide or control social engagement, such as avoiding eye contact or hiding one’s face. Social anxiety can contribute to avoidance behaviors. Those suffering from social anxiety are prone to overestimate the level of fear they will experience, and previous research has emphasized the predictive role of fear, with overpredictions of fear (Rachman and Lopatka, 1986; Rachman, 1994) motivating avoidance behaviors. It is likely that wearing a mask serves the dual function of preventing infections and masking obvious deficits in physical appearance or obvious signs of anxiety (Saint and Moscovitch, 2021). Therefore, it is further hypothesized that mask-wearing behavior may be driven by avoidance-related thoughts or behaviors, and therefore individuals may show a stronger intention to wear masks due to motivations of avoidance. Therefore, this study proposed the following hypotheses:

*H2*: Social anxiety positively influences the avoidance behaviors of college students;

*H3*: Avoidance positively influences mask-wearing intentions, and avoidance mediates the relationship between social anxiety and mask-wearing intentions.

On the other hand, past research on the effects of NCP has focused on understanding the nature of excessive fear or anxiety responses(overreactions) (Ahorsu et al., 2020; Jungmann and Witthöft, 2020; Tang et al., 2020). The Cognitive Behavioral Model of Health Anxiety (Taylor et al., 2004) proposes that beliefs are important determinants of emotions and health-related behaviors. Some research on past epidemics suggested that anxiety or lack of anxiety is an important driver of behavior (Taylor, 2022). Individuals who are overly anxious are likely to engage in socially disruptive behaviors (e.g., panic buying), may overly avoid social interactions, and may even stay at home for fear of being infected (Asmundson and Taylor, 2020; Taylor and Asmundson, 2020; Taylor, 2022). Thus, when people are fearful of COVID-19, they may show avoidance behaviors such as reducing use of public transportation, wearing masks or reducing mobility (Ito et al., 2022) in order to inhibit the spread of infectious diseases. In addition, recent research has shown that avoidance-based affective states are associated with stricter adherence to health behaviors (Wendel and Gable, 2023). On the basis of their fear of COVID-19, people are more inclined to wear masks to avoid infection. Therefore, this study proposed Hypothesis:

*H4*: Fear of COVID-19 positively predicts avoidance behaviors in college students, which indirectly predicts mask-wearing intentions.

#### 1.2.2 Impression management

Unlike avoidance behaviors, impression management primarily manifests strictly monitoring and controlling one’s behavior to attempt to create a favorable social image (Hirsch et al., 2004; Plasencia et al., 2011). Impression management behaviors are often viewed as adaptive behaviors in which individuals promote social connections by mimicking pro-social behaviors. However, in the process, these behaviors are still actually used in response to perceptions of threats, which can lead to persistent negative perceptions (Foa et al., 1996; Hofmann, 2004), thus entering a malignant cycle that further aggravates social fears. When the behaviors of impression management cannot be effectively controlled, there may be an increase in negative perceptions or a persistent fear of negative evaluations, further contributing to more severe avoidance behaviors, thus enhancing the intention to wear masks. Therefore, this study proposed the following hypotheses:

*H5*: Social anxiety positively regulates impression management;

*H6*: Impression management positively influences avoidance, and impression management may mediate between social anxiety and avoidance.

#### 1.2.3 Self-identity

Self-identity refers to the perception and internalization of an individual’s identification with his or her role (Rousseau, 1998). Self-concept formation, also known as personal identity formation, includes acceptance of physical changes, development of social and emotional competencies, and self-efficacy; self-concept deficits are characterized by low self-esteem, unclear descriptions of self, and difficulties with social roles, values, and choices, among other things (Erikson, 1968). The core features of SA are negative self-perceptions and fear of negative judgments (Clark and Wells, 1995; Rapee and Heimberg, 1997). Thus, low self-identity, that is, low acceptance of one’s role and negative or inadequate self-perception, so higher SA may predict lower self-identity. In a study by Hirsch et al. (2003), a link was found between negative self-image and increased anxiety, particularly among social anxious individuals. Cognitive models of SA emphasize the maintenance of the self in such situations and the role of the self in the etiology of the condition (Clark and Wells, 1995; Rapee and Heimberg, 1997; Hofmann, 2007; Stopa, 2009). Negative self-appraisals as a result of social anxiety may lead to lower self-identity, resulting in greater avoidance and avoidance of negative appraisals. A transient reduction in anxiety may be achieved by wearing masks.

According to Clark and Wells’ (1995) model, socially anxious individuals turn their attention to themselves in social situations and monitor themselves closely, which prevents them from detecting others’ reactions to them; physical sensations of anxiety and anxious mental states make it easy for them to use these feelings as confirmation of their negative beliefs. This further exacerbates self-focus, which may increase symptoms of fear and interfere with social interactions (Leigh et al., 2021). Furthermore, Erikson (1968) proposed that self-identity is a process of maturation along a continuum that involves the integration of an individual’s development of personality, which is primarily formed during youth. Meanwhile, during adolescence, neurocognitive changes support the development of key social and cognitive competencies such as self-awareness, the management of emotional expression, regulating emotion and sensitivity to social exclusion (Blakemore and Choudhury, 2006; Burnett et al., 2011; Haller et al., 2015). The development of these social and cognitive competencies are likely to influence the performance of safety behaviors (Leigh and Clark, 2018). Higher levels of self-identity increase both individuals’ motivation to play roles and the likelihood that they will actively do so (Li et al., 2022); conversely, low self-identity (i.e., an unstable sense of self and a lack of awareness of one’s own social roles) may lead to abandoning playing roles in interpersonal interactions and may lead to self-concealment, i.e., avoidance behaviors. Therefore, this study proposed the following hypotheses:

*H7*: Social anxiety negatively moderates self-identity. *H8*: Self-identity negatively influences avoidance behavior, and self-identity mediates between social anxiety and avoidance.

### 1.3 Purpose of the study

**Figure 1:**
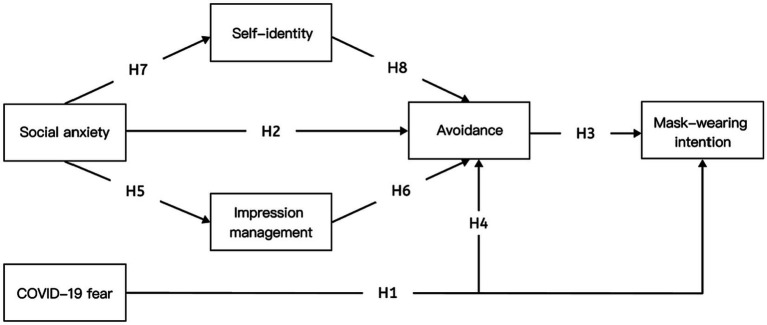
Research model.

In this study, we constructed a structural equation model (SEM) to systematically explore the relationship between college students’ fear of COVID-19 and mask-wearing intentions, and to explore the effects of social anxiety on self-identity, impression management, avoidance, and mask-wearing intentions, with the intention of exploring the differences between the two mechanisms. On the basis of previous studies, we proposed a theoretical model containing eight hypotheses in five pathways, (1) fear of COVID-19 directly and positively affects college students’ mask-wearing intentions (H1); (2) fear of COVID-19 positively affects college students’ mask-wearing intentions, with avoidance playing an indirect mediating role (H3, H4); (3) social anxiety positively affects college students’ mask-wearing intentions, with avoidance playing a mediating (H2, H3); (4) social anxiety positively affects college students’ mask-wearing intentions, and impression management and avoidance play a mediating role between social anxiety and intention (H3, H5, H6); (5) social anxiety positively influences college students’ mask-wearing intentions, social anxiety negatively moderates self-identity, self-identity negatively influences avoidance, and self-identity and avoidance mediate the relationship between social anxiety and intention to wear masks (H3, H7, H8) (Figure 1).

## 2 Materials and methods

### 2.1 Participants

A questionnaire was distributed at Guangdong University of Technology to recruit subjects, who were screened for self-reported social anxiety with scores greater than 30 on the Liebowitz social anxiety scale. This work has been approved by the Departmental Ethics Committee and the Institutional Review Board of the Guangdong University of Technology (No. GDUTXS2023123). All the participants provided informed consent before participation.

In total, 422 questionnaires were collected, and after excluding invalid questionnaires (e.g., those with regular responses and inattentive responses), subjects with self-reported social anxiety scores greater than 30 were screened, and finally 223 valid questionnaires were returned. The mean age of all subjects was 22.07 years (SD = 2.04), with 59 male and 164 female subjects.

### 2.2 Measures

#### 2.2.1 COVID-19 fear questionnaire

The COVID-19 fear questionnaire was developed by Chen and Lei (2022). In this study, the Chinese version of the COVID-19 Fear Scale (Feng et al., 2021) was used to assess the participants’ fear of COVID-19 (e.g., “I am most afraid of coronavirus-19″). The scale consists of 7 items and is rated on a Likert-5 scale ranging from 1 (not at all compliant) to 5 (fully compliant), with higher scores indicating higher levels of fear. The scale had good internal consistency with a Cronbach’s α of 0.85.

#### 2.2.2 Liebowitz social anxiety scale

The Liebowitz social anxiety scale (LSAS) was developed by Liebowitz (1987) to aid in the assessment of social anxiety disorders. The LSAS was originally conceptualized as a clinician-administered rating scale, but has since been validated as a self-reporting scale (Rytwinski et al., 2009). It contains ratings of fear and avoidance for 24 items on a Likert-4 scale ranging from 0 (never) to 3 (often). All fear and avoidance scores are summed to give a total score ranging from 0 to 144. Rytwinski et al. (2009) found that individuals with LSAS scores below 30 were less likely to develop SAD, so a cutoff score of 30 was used to screen valid subjects in this study. The scale has excellent internal consistency (Cronbach’s *α* = 0.94). The study used the social fear subscale (e.g., “Calling in public-your level of fear or anxiety”) for analyzing the data.

#### 2.2.3 Avoidance and impression management

Avoidance and impression management subscale was taken from the social behavior questionnaire (SBQ), which is primarily used to measure the use of safety behaviors in a range of social situations (Clark et al., 1995). The scale consists of 28 items on a 4-point Likert-type scale ranging from 0 (no safety behaviors) to 3 (very frequent safety behaviors), with higher scores indicating more frequent safety behaviors. The entries in the SBQ are divided into two types (Evans et al., 2021): 8 items for impression management (e.g., “Trying to get my words right”) and 13 items for avoidance (e.g., “Trying not to draw attention to myself”). The scale has good internal validity and good internal consistency (Total: Cronbach’s α = 0.91; Avoidance: Cronbach’s α = 0.86; Impression Management: Cronbach’s α = 0.87).

#### 2.2.4 Self-identity scale

The self-identity scale was developed by Oakes and Prager based on Erickson’s theory (Burger, 2000). The scale consists of 19 items (e.g., “I do not know what kind of person I am”) scored on a 4-point Likert-type scale ranging from 1 (not at all) to 4 (very much). Twelve of the questions are reverse-scored for the analysis. The higher the score, the higher the self-identity. The scale has good internal consistency (Cronbach’s *α* = 0.80).

#### 2.2.5 Mask-wearing intentions

The questionnaire provided pictures of relevant scenarios on campus and types of events (e.g., scenarios such as classes, group discussions), and the subjects were asked to imagine scenarios in which they would be active on campus and then to answer a question to indicate mask-wearing intentions (“Would you still wear a mask when you are active in the campus environment?”) The scale ranged from 1 (absolutely not) to 10 (absolutely would), with higher scores indicating greater mask-wearing intentions.

## 3 Results

### 3.1 Analyses of reliability and validity

The results of assessing the validity are detailed in Table 1. For convergent validity, the variables exhibited average variance extracted (AVE) values surpassing the threshold of 0.50, accompanied by composite reliability (CR) coefficients ranging from 0.76 to 0.87, all of which exceeded the critical value of 0.70. This underscores that the condition of convergent validity was met. In the domain of discriminant validity the square roots of the AVE values for each variable consistently exceeded the magnitudes of the correlation coefficients among the variables. This substantiates the successful achievement of the benchmarks of discriminant validity.

**Table 1:** Mean, standard deviation, the correlation matrix of each variable, convergent validity, and discriminant validity (*n* = 223).

	*M*	SD	CR	AVE	1	2	3	4	5	6
1. Social anxiety	1.46	0.43	0.83	0.51	–					
2. Avoidance	1.35	0.55	0.77	0.53	0.69***	–				
3. Impression management	1.86	0.65	0.87	0.52	0.53***	0.63***	–			
4. Self-identity	2.69	0.38	0.76	0.62	−0.45***	−0.45***	−0.28***	–		
5. COVID-19 fear	1.85	0.83	0.84	0.52	0.21**	0.25***	0.25***	−0.15*	–	
6. Mask-wearing intention	6.10	2.50	–	–	0.19**	0.28***	0.16*	−0.19**	0.25***	–

*n* = 223; *p < 0.05, **p < 0.01, ***p < 0.001. M, Mean; SD, Standard deviation; CR, Composite reliability; AVE, Average variance extracted.

Pearson’s correlation analysis was used to explore the correlations among social anxiety, impression management, avoidance, self-identity and mask-wearing intentions. The results in Table 1 show that social anxiety was significantly positively correlated with avoidance, impression management, and mask-wearing intentions, and significantly negatively correlated with self-identity; avoidance, impression management, and mask-wearing intentions were significantly positively correlated with each other; and self-identity was significantly negatively correlated with mask-wearing intentions. In addition, fear of COVID-19 was significantly positively associated with mask-wearing intentions and avoidance. A good statistical foundation was laid for the subsequent tests of the hypotheses.

### 3.2 Structural model and test of the hypotheses

We constructed a structural equation model (SEM) using AMOS 24.0 to test the hypothesized model framework and to explore the relationship between each variable and individuals’ mask-wearing intentions. Firstly, the model’s fit was tested, with CMIN/DF = 1.52, CMIN = 3372.79, DF = 2,456, CFI = 0.796, TFI = 0.788, and RMSEA = 0.048, indicating that the model’s fit was good.

**Figure 2:**
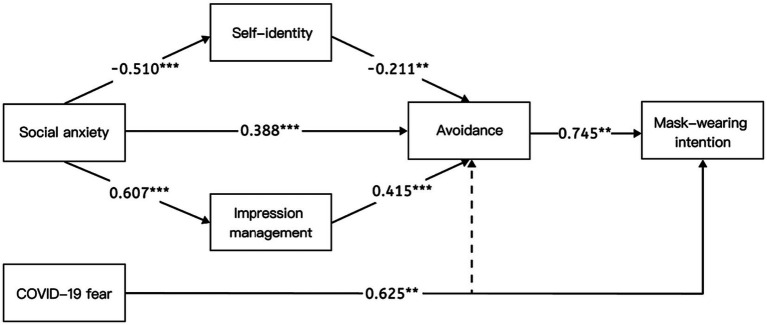
Results of the structural model. **p < 0.01, ***p < 0.001.

The interrelations among the variables were expressed as path coefficients, which effectively gauged the magnitude of influence between distinct variables. Positive coefficients signified positive correlations, while negative coefficients signified negative correlations. A comprehensive depiction of the parameters’ estimates and the hypotheses for the model of college students’ intentions to wear masks is presented in Table 2. The findings validate a substantial number of the hypotheses, further fortified by robust support from the amassed subject data. Specifically, our proposed model of college students’ intentions to wear masks was approached from two perspectives: psychological factors and social factors. On one hand, the study clearly confirmed the direct effect of the fear of COVID-19 on the intention to wear masks (H1, *β* = 0.625, *p* = 0.005). On the other hand, social anxiety indirectly affected college students’ intention to wear masks through the mediating role of avoidance (H2, *β* = 0.388, *p* < 0.001; H3, *β* = 0.745, *p* < 0.001), and the mediating role of impression management was verified (H5, *β* = 0.607, *p* < 0.001; H6, *β* = 0.415, *p* < 0.001), as was and the mediating role of self-identity (H7, *β* = −0.510, *p* < 0.001; H8, *β* = −0.211, *p* = 0.006). Notably, the fear of COVID-19 did not emerge as a significant predictor of avoidance (H4, *β* = 0.059, *p* > 0.05), unlike social anxiety. Detailed results are shown in Table 2; Figure 2.

**Table 2:** Structural model results.

Dependent variable	Hypothesis	Path	*β*	*p* value	Hypothesis supported
MWI	H3	Avoidance→MWI	0.745	0.001**	Supported
	H1	COVID-19 → MWI	0.625	0.005**	Supported
Avoidance	H2	SA → avoidance	0.388	<0.001***	Supported
	H4	COVID-19 → avoidance	0.059	0.271^n/s^	Not Supported
	H6	IM → avoidance	0.415	<0.001***	Supported
	H8	Self-identity→avoidance	−0.211	0.006**	Supported
IM	H5	SA → IM	0.607	<0.001***	Supported
Self-identity	H7	SA → self-identity	−0.510	<0.001***	Supported

**p < 0.01; ***p < 0.001; n/s, not significant. MWI, Mask-wearing intention; SA, Social anxiety; IM, Impression management.

### 3.3 Test of the mediating effects

This study used the bootstrap analysis technique by running model6 in PROCESS to conduct an analysis of the mediated effects to test the mediating roles of avoidance, impression management, and self-identity in the relationship between social anxiety and mask-wearing intentions, controlling for gender, age, and fear of COVID-19. The results of the regression analyses (see Table 3) showed that social anxiety was able to significantly and positively predict impression management (*β* = 0.71, *p* < 0.001) and avoidance (*β* = 0.53, *p* < 0.001), while social anxiety was able to significantly and negatively predict self-identity (*β* = −0.39, *p* < 0.001) among college students. Avoidance, as a mediating variable, was able to positively predict college students’ mask-wearing intentions (*β* = 1.13, *p* < 0.05), whereas impression management significantly and positively predicted avoidance (*β* = 0.30, *p* < 0.001), and self-identity significantly and negatively predicted avoidance (*β* = −0.23, *p* < 0.01).

**Table 3:** The mediating role of self-identity, impression management, and avoidance between social anxiety and mask-wearing intention.

	Self-identity			Impression management			Avoidance			Mask-wearing intention		
	*b*	*se*	*t*	*b*	*se*	*t*	*b*	*se*	*t*	*b*	*se*	*t*
Constant	3.30	0.09	37.60^***^	0.81	0.38	2.12^*^	0.59	0.25	2.33^*^	5.36	1.70	3.14^**^
Social anxiety	−0.39	0.05	−7.16^***^	0.71	0.10	7.45^***^	0.53	0.07	7.51^***^	−0.16	0.52	−0.30
Self-identity				−0.07	0.11	−0.69	−0.23	0.07	−3.29^**^	−0.47	0.48	−0.97
Impression management							0.30	0.04	6.81^***^	−0.22	0.33	−0.69
Avoidance										1.13	0.45	2.51^*^
*R* ^2^	0.59			0.30			0.21			0.12		
*F*	78.64			31.45			28.81			5.86		

*p < 0.05, **p < 0.01, ***p < 0.001; se, Standard error.

The bootstrap method was used to repeat the sampling process 5,000 times to calculate the 95% confidence intervals for the results of the mediation effect analysis and the mediation path diagram (see Table 4; Figure 2). Specifically, the mediating effect consisted of indirect effects in three pathways: Indirect Effect 1 acted through the pathway of social anxiety → avoidance → mask-wearing intentions; Indirect Effect 2 acted through the pathway of social anxiety → self-identity → avoidance → mask-wearing intentions; and Indirect Effect 3 acted through the pathway of social anxiety → impression management → avoidance → mask-wearing intentions.

**Table 4:** Bootstrap 95% confidence interval of mediating effect path.

	Effect	BootSE	BootLLCI	BootULCI	Relative mediation effect
Ind1: SA → avoidance → MWI	0.51	0.25	0.06	1.06	64.16%
Ind2: SA → self-identity → avoidance → MWI	0.09	0.05	0.01	0.22	10.87%
Ind3: SA → IM → avoidance → MWI	0.21	0.10	0.03	0.45	26.31%

Boot SE, Bootstrap standard error; Boot LLCI, Bootstrap lower limit confidence interval; Boot ULCI, Bootstrap upper limit confidence interval; MWI, Mask-wearing intention; SA, Social anxiety; IM, Impression management.

## 4 Discussion

Social anxiety is common among college students (Peng, 2004). Social anxiety can have a negative impact on several aspects of life, including performance, interpersonal communication, and psychological status. The removal of regulatory measures after COVID-19 may be particularly distressing for people with high social anxiety, as they face increased uncertainty and fear (Saint and Moscovitch, 2021). In this study, the effects of fear of COVID-19, social anxiety, avoidance, impression management, and self-identity on mask-wearing intentions were comprehensively examined by structural equation modeling using college students as the study population. This study revealed the effect of social anxiety on mask-wearing intentions and its internal mechanism of action, and also explored the differences between this effect and the effect of the fear of COVID-19 on mask- wearing intentions. The results of this study help us to understand the mechanism of social anxiety on the willingness to wear masks, to explore the psychological factors of whether and why socially anxious college students choose to wear masks, and to provide valuable guiding suggestions for safeguarding the physical and mental health of college students as well as adapting to the post-COVID-19 era.

### 4.1 The effect of the fear of COVID-19 on mask-wearing intentions

This study found that the fear of COVID-19 was significantly and positively associated with college students’ mask-wearing intentions, a result that demonstrates that in the post-COVID-19 period, people still choose to wear masks because of the perceived protection they provide. This finding is consistent with previous research. People reported that the primary motivation for choosing to wear masks was to protect themselves and others (Binter et al., 2023) from viral transmission and exposure. COVID-19, as an infectious disease, can lead to psychological distress, depression, anxiety, and fear (Duong, 2021; Satici et al., 2021; Lee and Crunk, 2022). The reason for the ability of fear of COVID-19 to directly predict mask-wearing intentions may lie in people’s perceptions of the risk of COVID-19. The degree of worry or fear associated with the threat of the disease is an important component of the perceived risk, while this aspect may be a strong motivator for engaging in certain behaviors or behavioral change (Crosby et al., 2001). Studies have shown a link between disease-related worry and behavioral intentions (Crosby et al., 2001; Sales et al., 2009). Fear of COVID-19 is also associated with following social distance guidelines (Broomell et al., 2020; Taylor et al., 2020; Harper et al., 2021). Thus, when college students still perceive the threat of COVID-19 in their daily socialization, they are more likely to choose to wear masks to ward off illness and maintain good health.

### 4.2 Influence of social anxiety on mask-wearing intentions

This study found that college students’ social anxiety was significantly and positively correlated with their mask-wearing intentions, and the results proved the effect of social anxiety on mask-wearing intentions, namely that socially anxious people may choose to wear masks due to their fear of exposing their appearance or their desire to hide themselves in a group, which is in line with the results of a previous study (Saint and Moscovitch, 2021). However, the direct predictive effect of social anxiety on mask-wearing intentions was not significant. The likely reason for this is that fear of COVID-19 was significantly positively correlated with mask-wearing intentions in the study, and college students may have perceptions of the consequences of contracting COVID-19 and of the protective function of masks, and thus choose to wear a mask (Nakayachi et al., 2020). Further, wearing a mask obscures a major portion of the face, which includes elements that provide key information about an individual’s identity (e.g., trustworthiness, attractiveness, age, and gender), and face masks can have a significant impact on social interactions (Bruce and Young, 1986). Because a face mask is an obstacle to accurately recognizing facial expressions and evaluating emotions (Bassili, 1979), socially anxious college students may worry about the uncertainty caused by others’ misinterpretation of their facial emotions, and fear being observed and judged, which can lead to adverse social experiences. As a result, they may be more inclined not to wear a mask during social interactions. It can be seen that willingness to wear a mask may be associated with differences in social anxious college students’ assessment of situations, which, in turn, indirectly affects their mask-wearing intentions.

#### 4.2.1 Mediating role of avoidance

In the present study, we found that avoidance acted as a common mediator of the three indirect effects and positively predicted college students’ mask-wearing intentions. Hirsch et al. (2004) showed that avoidance behaviors were associated with negative perceptions of observers, whereas impression management behaviors were not. In addition, several studies have shown that high levels of social anxiety significantly increase the risk of increased negative self-perception, and that the fear of being negatively evaluated in social situations, in turn, can lead to high levels of interpersonal distress and avoidance (Clark and Wells, 1995; Rapee and Heimberg, 1997; Hofmann, 2007; Moscovitch, 2009). Therefore, for those who are concerned about being negatively evaluated by others, wearing a mask can be viewed as a form of self-concealment that enhances comfort and reduces anxiety in social interactions (Saint and Moscovitch, 2021). A possible mechanism by which social anxiety indirectly affects mask-wearing intentions is that college students may have more severe negative thoughts after evaluating a social interaction, which leads to a greater tendency to choose to avoid negative evaluations by avoiding them in order to alleviate social tension. They may choose to continue to wear masks as an avoidance strategy, so their mask-wearing intentions will be stronger.

In conclusion, avoidance mediates the relationship between social anxiety and mask-wearing intentions, which may be because individuals tend to avoid negative evaluations and social stress by wearing a mask, thus enhancing mask-wearing intentions.

#### 4.2.2 Mediating role of impression management and self-identity

Furthermore, our study revealed an association between social anxiety and impression management, which is consistent with the assumptions of the cognitive–behavioral model of SA (Clark and Wells, 1995). According to this model, safety behaviors are used as a means of preventing or minimizing the feared outcomes. Thus, the reason why social anxiety in college students positively predicted impression management but ultimately influence mask-wearing intentions through avoidance may be that the cognitive–behavioral model of SA has long emphasized the negative effects of safety behaviors, and research has shown that the use of safety behaviors actually impairs social performance while increasing perceptions of anxiety and negative social outcomes (Plasencia et al., 2011; Moscovitch et al., 2013; Rowa et al., 2015), which is a maintaining factor of social anxiety (Wong and Rapee, 2016). As social anxiety increases, individuals may face more severe outcomes. In this case, college students may practice self-concealment to reduce the perceived social threat, and wearing a mask will make them feel safer and reduce social distress.

Research has also found that self-identity mediates the relationship between social anxiety and avoidance among college students. In the cognitive–behavioral model of SA (Clark and Wells, 1995), it was argued that socially anxious individuals tend to develop negative self-thoughts as a result of early unpleasant experiences, which leads to negative perceptions in social situations. They turn their attention to themselves and infer others’ perceptions of themselves from information within themselves (Cheng et al., 2015; Ran et al., 2018), thus developing a poor self-image. The college students with high social anxiety in this study had relatively low self-reported self-identity, which is consistent with previous research (Gao and Hu, 2021). Additionally, the reason that social anxious of college students reversely predict self-identity but ultimately influence intentions to wear a mask through avoidance may be that the basis for the assumed relationship between self-identity and behavioral intentions relies on identity theory (Stryker, 1968, 1987). Terry et al. (1999) argued that self-identity influences intentions because performing a behavior allows the individual to come to validate the self-concept derived from role identity and helps the individual to develop a positive and significant self-evaluation. Because of their perceived low self-image, social anxiety groups are more inclined to engage in avoidance behaviors and to engage in behaviors that are perceived to be more in line with personal norms or social roles (wearing masks to avoid interactions). Another point in the results that was consistent with existing research is that when they have a poor self-image, individuals may believe that others are evaluating them in the same way, thereby producing a range of expressions such as nervousness, lowering the head and gaze avoidance (Cheng et al., 2015). The psychological barriers brought about by low self-identity will further affect individuals’ behaviors in daily social situations, and college students are very likely to choose to avoid social interactions. Even if masks are no longer required in the post-COVID-19 era, these college students may still choose to continue using masks as an avoidance strategy.

### 4.3 Research implications and limitations

Our findings make important contributions to theory and practice, and it is worth mentioning that while the existing literature has mainly focused on mask-wearing intentions in the context of health behaviors during epidemics, the present study takes a new perspective by exploring the motivations of individuals to use masks outside of health behaviors, and by analyzing and investigating the differences in the mechanisms of these two different motivations.

In summary, this study revealed two mechanisms that influence mask-wearing intentions. The first is the direct mechanism, through which fear of COVID-19 affects mask-wearing intentions, and the second is the internal mechanism, through which social anxiety affects mask-wearing intentions, revealing the psychological influences on college students’ choices about mask-wearing in the post-epidemic era. First, on the one hand, the high risk of post-pandemic post-traumatic stress disorder, as demonstrated by Cavalera et al. (2023), suggests that COVID-19 has had a dramatic impact on individual’s mental health; Focusing on the negative self-conscious emotions and counterfactual thoughts associated with the trauma of COVID-19 fears or related experiences may provide answers to questions about individual’s willingness to wear masks in the post- COVID-19 era. On the other hand, it’s also some theoretical grounding is provided for how college students with social anxiety may be able to comply with the trend to remove their masks. Although the use of self-concealment strategies may allow them to feel safer and more protected in social situations, this tends to impair social performance and reduce the quality of social interactions (Rowa et al., 2015). Low-quality social interactions may lead college students to develop negative memories of social situations and thus choose to avoid them in all future interactions, creating a vicious cycle in which the symptoms of social anxiety are not alleviated or improved. Therefore, the teachers and parents should pay more attention to students’ psychological state, be aware of the impact of post-traumatic stress disorder caused by negative experiences of the pandemic on life, and promptly solve the psychological problems the students may have in facing social situations. In addition, removing masks may also be associated with existing exposure therapies for treating social anxiety, and attempting to remove a mask may imply the abandonment of safety behaviors, which ca significantly improve the symptoms of social anxiety in the course of treatment (Cougle et al., 2020). Of course, future research needs to further explore how safety behaviors, including mask-wearing, can be improved to facilitate the effective implementation of exposure therapy. In addition, focusing on the feature of masks shielding the face, there have been studies to explore the use of masks for normal people (Carbon, 2020; Miyazaki et al., 2022) and those with severe mental illness (Bulgari et al., 2020) pose challenges in interpreting the emotions of others, and future research should more fully explore the social impact of mask wearing on other vulnerable groups, including those with social anxiety as presented in this study.

Nonetheless, there are some limitations to this study. Firstly, the study used a self-reporting approach, and college student subjects may not be able to report clinically true emotions or behaviors. Secondly, the selection sample is narrow and consists only of college students. Moreover, this study used a cross-sectional research method, the predictive effects of the main variables were based on existing theoretical assumptions, and temporal causality was not been verified. This is expected to be confirmed in a larger sample size and other subjects in China in the future and to also reveal the behavioral basis of the association of mask-wearing intentions and other factors further with social anxiety through more measures (such as EEG, fMRI and other tools to measure anxiety), to develop more targeted interventions.

## 5 Conclusion

In this study, we constructed a model of college students’ mask-wearing intentions that included the factors of fear of COVID-19, social anxiety, self-identity, impression management and avoidance. It was found that fear of COVID-19 positively predicted mask-wearing intentions; college students’ social anxiety positively predicted self-identity, avoidance and impression management; social anxiety positively predicted avoidance through the mediating effect of impression management; social anxiety negatively predicted avoidance through the mediating effect of self-identity; and avoidance acted as a common mediator to positively predict college students’ mask-wearing intentions. Our study provides important insights into understanding the mechanisms by which both fear of COVID-19 and social anxiety influence mask-wearing intentions in the post-epidemic context.

## Data availability statement

The raw data supporting the conclusions of this article will be made available by the authors, without undue reservation.

## Ethics statement

The studies involving humans were approved by the Departmental Ethics Committee and the Institutional Re-view Board of the Guangdong University of Technology (No. GDUTXS2023123). The studies were conducted in accordance with the local legislation and institutional requirements. The participants provided their written informed consent to participate in this study.

## Author contributions

TX: Conceptualization, Writing – original draft. XX: Conceptualization, Investigation, Methodology, Writing – original draft. SD: Conceptualization, Supervision, Writing – review & editing.
